# Correction: Case Report of a patient with Madelung's disease combined with alcoholic liver disease and liver cirrhosis

**DOI:** 10.3389/fmed.2026.1810907

**Published:** 2026-03-04

**Authors:** Frontiers Production Office

**Affiliations:** Frontiers Media SA, Lausanne, Switzerland

**Keywords:** Madelung's disease, liver cirrhosis, alcohol consumption, metabolic disorder, case report

An incorrect DOI (10.3389/fmed.20251695921) was registered for this article on publication. The correct DOI (10.3389/fmed.2025.1695921) has now been registered.

The original version of this article has been updated.

